# Sustainable development of an effective anti-corrosion film over the St12-steel surface against seawater attacks using Ce(III) ions/tri-sodium phosphate anions

**DOI:** 10.1038/s41598-023-38540-9

**Published:** 2023-07-27

**Authors:** Farshad Bahremand, Taghi Shahrabi, Bahram Ramezanzadeh, Seyed Ali Hosseini

**Affiliations:** 1 Department of Materials Engineering, Faculty of Engineering, Tarbiat Modares University, P.O. Box 14115-143, Tehran, Iran; 2 Department of Surface Coatings and Corrosion, Institute for Color Science and Technology, Tehran, Iran

**Keywords:** Environmental impact, Materials science

## Abstract

One application of organic compounds is to utilize them as corrosion inhibitors in acidic environments to diminish steel corrosion. These inhibitors do not show very good inhibition properties in saline (NaCl) environments. There have been many studies on boosting these inhibitors’ performance in such environments (especially Cl^−^ containing media). One of the ways that have been proposed is the use of organic and inorganic inhibitors, simultaneously. The synergistic effect of these inhibitors has shown promising results in reducing steel corrosion. In this study, cerium(III) nitrate and tri-sodium phosphate (TSP) was used as organic and inorganic inhibitors to control the corrosion of steel in a 3.5 wt.% NaCl environment. The corrosion measurements were conducted in the 3.5 wt.% NaCl environment by EIS and polarization methods. Surface studies were done by SEM, Raman, GIXRD, and EDS methods. Corrosion studies (EIS and polarization) have revealed that when 500 ppm of Ce(NO_3_)_3_ and 500 ppm of TSP are added to the 3.5 wt.% NaCl medium, the highest synergism index (1.27) and inhibition efficiency (73.7%) are achieved. Also, by adding 500Ce-500TPS to the solution, *i*_corr_ and *R*_ct_ of steel decreased by about 80% and increased approximately 4-fold, respectively. This improvement in the steel performance against corrosion in the presence of an equal ratio of Ce(NO_3_)_3_ and TSP is the outcome of the formation of a hydrophobic dense film (consisting of Ce(OH)_3_, Ce/Fe-phosphate complexes) on the metal surface. This claim has been proven by SEM/EDS, contact angel, FT-IR, and XRD analysis.

## Introduction

The low resistance of St-12 type steel, a famous industrial grade alloy, against corrosion is one of the challenging dilemmas in its application^1–3^. Various methods are proposed to minimize this problem, such as anodic/cathodic prevention, the use of anti-corrosion coatings, and the application of inhibitors^4–6^. Among these, inhibitors have gained more heed today because of their ease of use and low cost. The efficiency of the inhibitors and their inhibition mechanism pertains to several parameters (such as corrosive ions, the chemical structure of inhibitors, the number of absorbed sites on the met-surface, etc.)^7^. Organic/inorganic are two primary groups of inhibitors according to their chemical structure^8^. Organic inhibitors usually form a barrier film on the metal surface. They have many functional groups, including electron-rich heteroatoms (O, N, and S) in their structure^9–11^. On the other hand, inorganic inhibitors can postpone the anodic/cathodic reactions. They are partitioned into two major groups, including anodic and cathodic, based on their significant impact on the anodic or cathodic reactions^12^. Organic inhibitors perform well in acidic environments; many researchers have worked hard to find an organic inhibitor that can delay the corrosion of steel in saline environments (such as 3.5 wt.% NaCl solution). On the other hand, inorganic inhibitors show good proficiency in saline mediums. Steel usually suffers from corrosion by saline solution attacks in water storage tanks, seawater transition pipelines, etc. In saline environments, chloride anions play a prominent role in steel corrosion. Organic corrosion inhibitors do not have good efficiency in the chloride-rich environments. So, combining the organic and inorganic inhibitors is an idea for overcoming this problem which has been seen in most studies, recently^13,14^.

Chromate and zinc compounds are anodic and cathodic inorganic inhibitors, respectively, which are superior corrosion inhibitors for various metals in neutral salt-contain solutions. However, due to the environmental hazards, their use has been strictly prohibited by various organizations^15–20^. Many organic and inorganic corrosion inhibitors have been employed as alternatives to chromates. It has recently been proven that lanthanide (rare earth elements, REE) compounds can be used as environmentally-friendly green corrosion inhibitors^21–24^. The Ce-based salts have been employed as corrosion inhibitors, successfully. These compounds have demonstrated good corrosion protection behavior at a concentration of about 1000 ppm (~ 2.68 mM)^25^. Cerium(III) compounds are cathodic inhibitors that can reduce the cathodic reaction rate, and block the cathodic sites via the formation of the Ce-oxide/hydroxide compounds^26,27^. Ce(III) compounds are not potent inhibitors because they cannot provide a dense and defect-free film over the surface of the metal.

A practical method to reach a high level of protection from the inhibitors is combining them to achieve a synergistic effect^28^. Ramezanzadeh et al.^29^ studied the effectiveness of Nettle leaf extract and Ce-nitrate mixtures against mild steel corrosion retardation. They revealed that a compact nanostructure film was formed on the steel surfaces by using the NLE-Ce inhibitors. Xio and coworkers^30^ have investigated the synergistic corrosion inhibition demeanor of Ce-ions & serine on the mild steel corrosion mitigation in a 3 wt.% sodium chloride solution. They concluded that a Ce–Ser complex film was developed on the steel surface. However, there are few reports of cooperation between REE compounds and inorganic inhibitors. The synergism between Ce(NO_3_)_3_ and sodium-dodecyl-benzene-sulfonate on the corrosion of AA5052 aluminum alloy in 3 wt.% NaCl media has been checked by Lie et al. and an 89.7% inhibition efficiency was concluded^31^.

Phosphate compounds are vastly utilized as corrosion inhibitors that can diminish the metals’ corrosion through anodic, cathodic, or mixed inhibition mechanisms^32^. Phosphates are eco-friendly and have high efficiency of corrosion inhibition^33^.

In our research, the synergistic inhibition impact of cerium(III) nitrate and phosphate anions toward mild steel corrosion in saline media was explored. For this aim, tri-sodium phosphate (TSP) and Ce(III) ions were used in three different mixing levels. Corrosion assessment was performed using EIS and the potentiodynamic polarization technique. In addition, the SEM, EDS, CA (contact angle), XRD, and FT-IR tests were done to inspect the deposited film morphology and composition.

## Experimental

### Raw materials

St-12 steel sheets with a chemical composition of 0.10 wt.% C, 0.035 wt.% S, 0.035 wt.% P, 0.007 wt.% N, 0.45 wt.% Mn and remaining Fe with 3 cm × 5 cm × 0.2 cm dimensions were used as the substrate. cerium(III) nitrate and tri-sodium phosphate dodecahydrate were purchased from Merck Co. and, NaCl was obtained from Mojallali Co. (Iran). For steel panels’ abrasion, SiC papers (from 220 to 800 grit size) have been utilized. The sheets were degreased with acetone and then rinsed in DW (distilled water). The solutions studied in this research are presented in detail in Table 1.

**Table 1:** Description of the solutions prepared in this study.

Abbreviation	Description
Blank	3.5 wt.% NaCl
1000Ce	3.5 wt.% NaCl + 1000 ppm cerium(III) nitrate
1000TSP	3.5 wt.% NaCl + 1000 ppm tri-sodium phosphate
750Ce-250TSP	3.5 wt.% NaCl + 750 ppm cerium(III) nitrate + 250 ppm tri-sodium phosphate
500Ce-500TSP	3.5 wt.% NaCl + 500 ppm cerium(III) nitrate + 500 ppm tri-sodium phosphate
250Ce-750TSP	3.5 wt.% NaCl + 250 ppm cerium(III) nitrate + 750 ppm tri-sodium phosphate

### Characterization

#### Corrosion properties appraisal

EIS, along with polarization analyses, were employed to check the corrosion performance of the uninhibited and inhibited specimens. For the measurements, the working electrode, saturated calomel reference electrode, and the Pt-electrode were employed. In this case, the uninhibited and inhibited specimens (1 cm^2^) were utilized as testing electrodes. This specified three-electrode cell was immersed in 3.5 wt.% NaCl solution at ambient temperature and neutral pH. It should be that the effect of temperature and pH on corrosion behavior can also be investigated, which was not checked in this study^34,35^. Electrochemical impedance spectroscopy was accomplished by Autolab PGSTAT 302N instrument at OCP, in 10^5^–10^–3^ Hz range with ± 10^–3^ V potential-perturbation at various plunging times for up to 72 h. In the following, Nova software was utilized to elicit the impedance parameters and, the experimental data were fitted via selected models using ZView software. To ensure the accuracy and repeatability of the obtained results, all evaluations were performed on three similar samples and, the average outcomes were reported. In the case of the polarization analysis, the cell form was precisely like the EIS test, and the experiment was performed after 72 h. The potential amplitude of ± 0.25 mV, close to the recorded OCP values, with a 0.06 V/min scan rate, was the test condition. As well, in this test, the repeatability of all evaluations was surveyed on three samples.

#### Surficial film analysis

The morphology and chemical composition of the films precipitated on the steel sheets (1 cm^2^), which were immersed in various solutions (Table 1) for 48 h, were investigated by SEM (model VEGA/TESCAN-XMU) and EDS (model Mira III/TESCAN-SAMX). The test was accomplished using a BE (backscatter electron) detector at a V = 15 kV. Furthermore, FT-IR spectroscopy (model Perkin-Elmer Frontier, USA/in 400–4000 cm^−1^ wavenumber range) and XRD test (model X’Pert MPD, Netherlands/from 5 to 85 degrees for 2θ with θ–2θ scan mode) were performed to evaluate the surface functional groups and different phases formed, respectively. In addition, a CA test was done by OCA 15 plus using 1 mL DW at T = 25 ± 2 °C to check the surface wettability of the samples. The appearance of the DW dripped on the samples was taken by a Canon model digital camera.

## Results and discussion

### Corrosion behavior evaluations

#### OCP measurements

**Figure 1:**
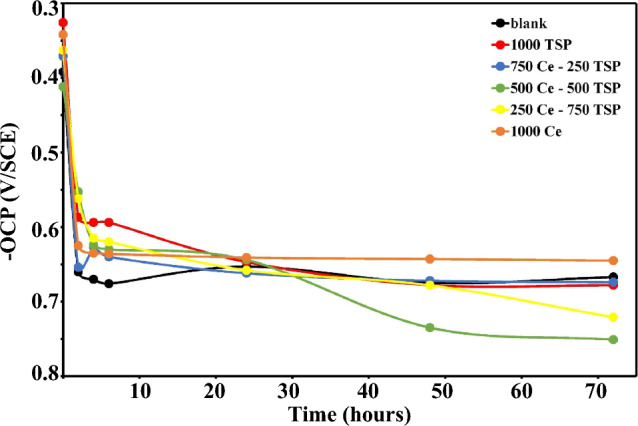
Open circuit potential (OCP) variations over immersion time for the steel coupons immersed in the 3.5 wt.% NaCl solutions without and with various concentration of Ce/TSP.

The OCP values recorded for the samples subjected to the 3.5 wt.% NaCl solutions (with/without inhibitors) were measured for up to 72 h and, plots were shown as a function of plunging time in Fig. 1. In the first minutes of immersion, OCP shows a significant decrement due to the corrosion reactions taking place on the steel-substrate. This reduction has a steeper slope in the blank specimen than in the inhibited samples. The amount of OCP over 8 h is approximately constant, indicating film formation on the surface and prevention of oxidation. The OCP value in the 500Ce-500TSP sample is more negative, demonstrating a reduction in the rate of the cathodic reaction due to Ce-phosphate deposition. In addition, in this sample, a steady-state condition was achieved compared to other samples in longer times, which indicates corrosive agents’ accesses limitation to the substrate due to the attendance of the dense film deposition on the surface.

#### Polarization analysis

**Figure 2:**
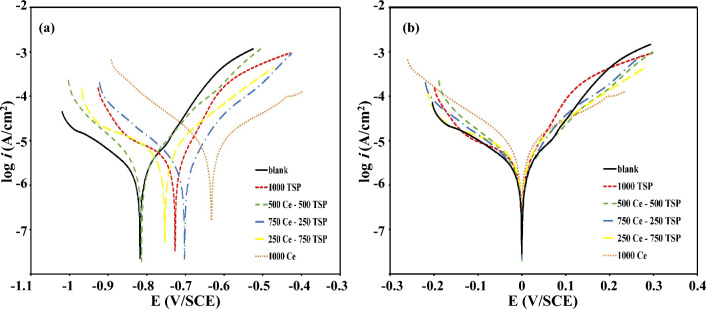
(**a**) Polarization plots of uninhibited and inhibited (with different concentration of Ce/TSP) samples immersed in 3.5 wt.% NaCl media for 72 h at 25 °C, (**b**) Shifted plots to *E*_corr_ = 0.

Polarization curves of the exposed samples to the untreated and treated solutions for 72 h were shown in Fig. 2. The electrochemical parameters were calculated from the polarization curves through the Tafel method, and data were given in Table 2.

**Table 2:** Electrochemical parameters obtained from the polarization curves for the steel samples immersed in the 3.5 wt.% NaCl solutions without and with various concentration of Ce/TSP.

Sample	*E*_corr_ (mV/SCE)	*i*_corr_ (µA cm^−2^)	*β*_a_ (V/dec)	− *β*_c_ (V/dec)	Efficiency (%)
Blank	− 816.08 ± 1%	13.81 ± 2%	0.28 ± 8%	0.15 ± 21%	–
1000TSP	− 725.33 ± 4%	5.70 ± 33%	0.41 ± 18%	0.11 ± 19%	58.70
1000Ce	− 632.96 ± 2%	11.07 ± 11%	0.19 ± 1%	0.20 ± 1%	19.80
750Ce-250TSP	− 711.43 ± 1%	3.26 ± 9%	0.15 ± 4%	0.09 ± 9%	76.40
250Ce-750TSP	− 755.92 ± 1%	7.88 ± 2%	0.40 ± 2%	0.16 ± 3%	42.90
500Ce-500TSP	− 813 ± 2%	2.79 ± 14%	0.13 ± 1%	0.11 ± 2%	79.70

*E*_corr_ corrosion potential, *i*_corr_ corrosion current density, *β*_a_ anodic branch Tafel slope, *β*_c_ cathodic branch Tafel slope.

Furthermore, to calculate the inhibition degree (*η*), Eq. (1) was used and, the results are shown in Table 2.

1 $$\eta\left( \% \right) = \left[ {\left( {i_{0} {-}i} \right)/i_{0} } \right] \times {1}00$$

where *η*, *i*, and *i*_0_ are the inhibition efficiency, and corrosion current density in the presence and absence of the inhibitors, respectively. According to the observations and analysis of Fig. 2, *E*_corr_ was altered to less negative values in the samples plunged in the inhibited solution. The polarization test results divulged that the *i*_corr_ of all samples was lower than the untreated sample, and the reduction in the cerium or phosphate inhibitors containing electrolytes was less than the mixed inhibitors (Ce + TSP). The synergistic effect is the reason for this further reduction. The shift of *E*_corr_ for 500Ce-500TSP was less than others, indicating that the inhibition mechanism is mixed type (cathodic and anodic type)^36^. Also, according to Table 2, 500Ce-500TSP has shown the maximum inhibition efficiency among all samples. The Ce^3+^ can be adsorbed on the cathodic sites and react with (OH^-^) ions, creating a Ce(OH)_3_-based protective film. However, Ce(NO_3_)_3_ is a mixed-type inhibitor because of the attendance of (NO_3_^-^)-ions which affect the anodic reactions too. Also, the phosphate anions affect the anodic reaction and can form a Fe-phosphate complex with Fe cations on the metal surface. Figure 2 shows that the *i*_a_/*i*_c_ of the treated samples shifted to lower values. The *β*_a_ & *β*_c_ were changed for mixing Ce and phosphate ions. These observations reveal that the corrosion and protection mechanism of both anodic/cathodic reactions were altered. The variation of Tafel slopes of the anodic and cathodic branches in the attendance of a mixture of inhibitors compared to the blank solutions demonstrates the synergistic effect of the inhibitors with mixed-type inhibition.

#### EIS analysis

**Figure 3:**
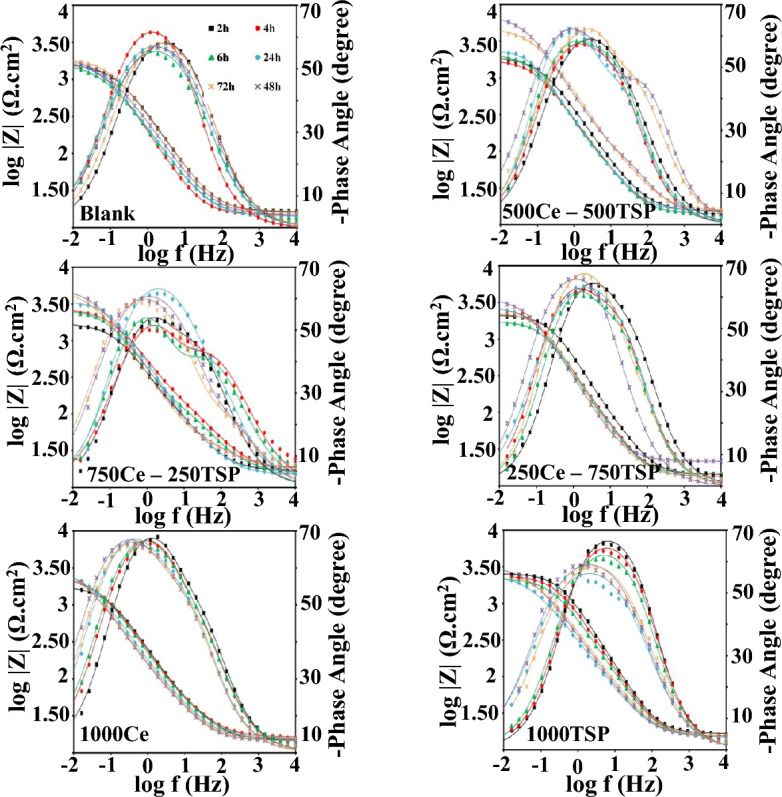
Bode plots of various samples immersed in 3.5 wt.% NaCl with/without inhibitors.

**Figure 4:**
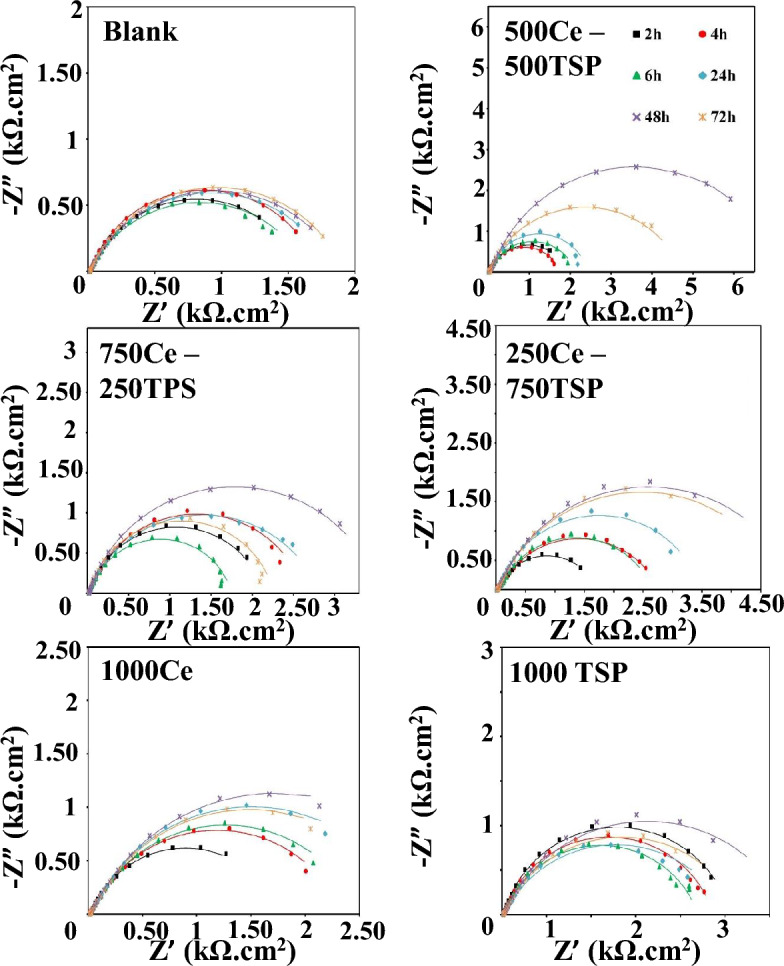
Nyquist plots of various samples immersed in 3.5 wt.% NaCl with/without inhibitors.

**Figure 5:**
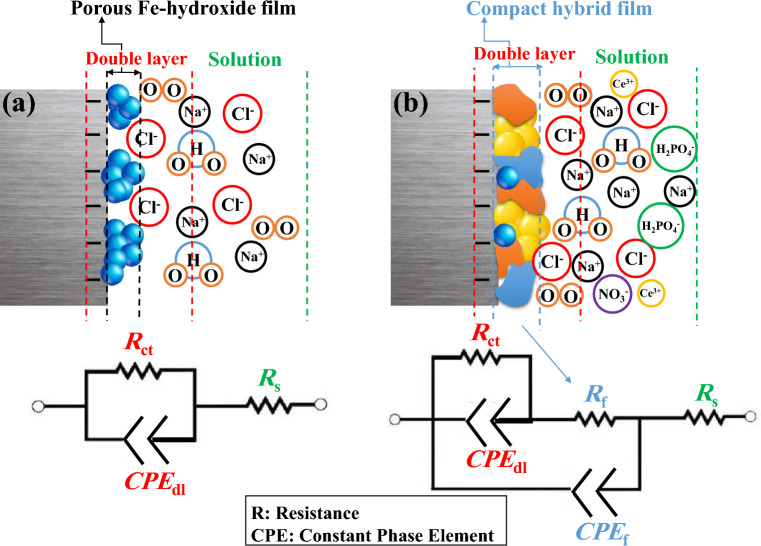
EEC (electrical equivalent circuits) (**a**) one time-constant, (**b**) two time-constant.

Electrochemical impedance spectroscopy measurements were done on the steel coupons plunged in the 3.5 wt.% NaCl medias comprising cerium cations and phosphate anions (as a mixture of both and sole) at different immersion times (T = 25 ± 1 °C). The Bode and Nyquist plots of the steel specimens exposed to various solutions are displayed in Figs. 3 and 4. The measurements were carried out for the test times of 2, 4, 6, 24, 48, and 72 h at around the OCP. As shown in Fig. 5, two EEC (electrical equivalent circuits) were employed to model the EIS data^37^. The porosity and non-uniformity of the surface layer have led to the use of constant phase elements as an alternative to the ideal capacity. The electrochemical parameters, extracted from each model are given in Tables 3 and 4. The *CPE*_dl_ is included *Y*_0_ and *n*.

**Table 3:** The electrochemical parameters extracted from impedance plots of the uninhibited and inhibited with 1000 ppm Ce and 1000 ppm TSP samples at room temperature immersed in 3.5 wt.% NaCl solution for different times.

Sample	Time (h)	*R*_ct_ (Ω cm^2^)	CPE		*R*_f_ (Ω cm^2^)	CPE		
			*Y*_0_ (μΩ^−1^ cm^−2^ s^n^)	*n*		*Y*_0_ (μΩ^−1^ cm^−2^ s^n^)	*n*	*η* (%)
Blank	2	1800	684	0.76	–	–	–	–
	4	1714	114	0.79	–	–	–	–
	6	1643	1124	0.72	–	–	–	–
	24	1823	1153	0.73	–	–	–	–
	48	1888	956	0.73	–	–	–	–
	72	1905	734	0.74	–	–	–	–
1000TSP	2	2548	219	0.83	–	–	–	29.30
	4	2360	280	0.81	–	–	–	27.40
	6	2186	356	0.79	–	–	–	24.90
	24	2527	840	0.70	–	–	–	27.60
	48	3229	689	0.73	–	–	–	41.50
	72	2675	609	0.73	–	–	–	28.80
1000Ce	2	1649	312	0.81	109	632	0.77	2.40
	4	2223	325	0.77	111	743	0.75	26.60
	6	2388	362	0.77	110	787	0.74	34.20
	24	2770	440	0.80	212	1077	0.74	38.90
	48	3263	635	0.75	159	1194	0.74	44.80
	72	2753	570	0.77	188	1163	0.74	35.20

*R*_ct_ charge transfer resistance, *CPE* constant phase element, *Y*_0_ admittance, *n* exponent, *R*_f_ film resistance, *η* inhibition efficiency.

**Table 4:** The electrochemical parameters extracted from impedance plots of the uninhibited and inhibited with various concentration of Ce/TSP samples at room temperature immersed in 3.5 wt.% NaCl solution for different times.

Sample	Time (h)	*R*_ct_ (Ω cm^2^)	CPE		*R*_f_ (Ω cm^2^)	CPE		
			*Y*_0_ (μΩ^−1^ cm^−2^ s^n^)	*n*		*Y*_0_ (μΩ^−1^ cm^−2^ s^n^)	*n*	*η* (%)
750Ce-250TSP	2	1367	187	0.89	331	501	0.74	6
	4	2449	152	0.85	332	327	0.67	38.40
	6	2423	237	0.88	242	362	0.69	38.30
	24	3418	186	0.80	62	256	0.8	47.60
	48	5119	349	0.76	31	225	0.76	63
	72	4885	439	0.76	37	262	0.75	61
250Ce-750TSP	2	1883	69	0.88	245	284	0.83	15.40
	4	2111	128	0.90	520	501	0.81	34.80
	6	1470	179	0.91	265	473	0.82	5.30
	24	2746	233	0.66	77	424	0.83	35.40
	48	3269	17	1.13	272	706	0.81	46.70
	72	2204	222	0.85	42	340	0.87	15.20
500Ce-500TSP	2	1792	111	0.76	125.80	522	0.76	6.10
	4	1164	212	0.88	572.60	773	0.76	1.30
	6	1496	212	0.9	625.50	742	0.76	22.60
	24	2272	329	0.88	150	533	0.81	24.70
	48	7036	262	0.78	127	149	0.83	73.70
	72	4288	27.5	1.04	466	342	0.73	59.90

*R*_ct_ charge transfer resistance, *CPE* constant phase element, *Y*_0_ admittance, *n* exponent, *R*_f_ film resistance, *η* inhibition efficiency.

**Figure 6:**
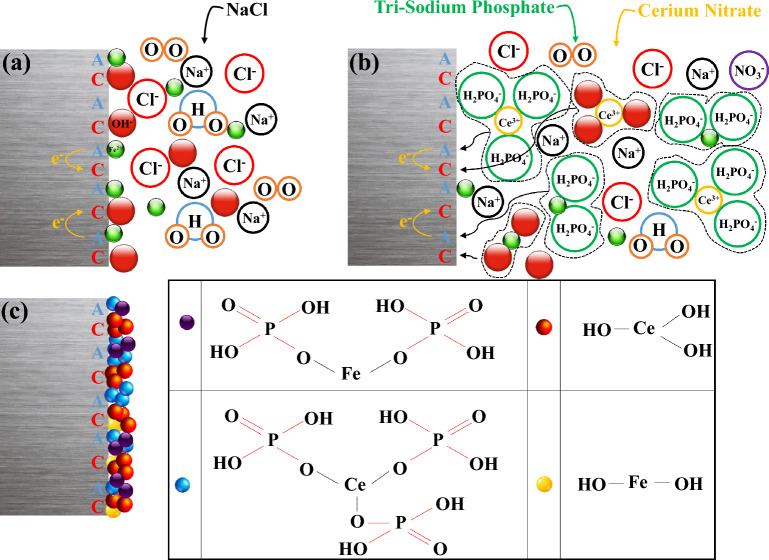
Schematic of the organic–inorganic complexes and uniform/compact film formation on the steel surface in the presence of two inhibitors.

According to Figs. 3 and 4, in the Nyquist & Bode plots of the blank and 1000 TSP samples, only a single-time constant can be seen, indicating that the occurrence of the electrochemical reaction on these samples is under the control of charge transfer (CT). Besides, for the other samples, a new time constant became visible at high *f*, exhibiting the formation of a compact inhibitor layer on the steel substrate. So, a double-time-constant electrical model is employed for modeling impedance data in these specimens (Fig. 5). As can be seen from Tables 3 and 4, by adding Ce/TSP inhibitor to the corrosive solution, the *R*_ct_ of the steel is increased (from 1823 to 2770 Ω cm^2^). This increment is even more remarkable when a mixture of two inhibitors is used (from 1823 to 7036 Ω cm^2^). With increasing the plunging time, the *R*_ct_ of the blank sample is constantly increased and decreased, indicating the formation/detachment of the corrosion products on/from the steel surface. The *R*_ct_ enhancement over time is also observed in the samples subjected to the solutions containing sole inhibitors (1000Ce and 1000TSP). The increment of the *R*_ct_ of the 1000Ce sample is greater than the 1000TSP one; in addition, in the 1000Ce sample, a two-time constant has appeared that demonstrates the production of a compact film on the substrate. The increase in *R*_ct_ over time is much more drastic in 250Ce-750TSP, 750Ce-250TSP, and 500Ce-500TSP samples, so that in all samples, after 48 h a significant enhancement in *R*_ct_ was observed. For example, in 500Ce-500TSP after 48 h of immersion, *R*_ct_ more than 7 kΩ cm^2^ was obtained. In addition, like 1000Ce, in these samples (inhibited with mixed inhibitor), a two-time constant revealed that a compact film was produced on the steel sheet. The fluctuation of the *R*_f_ values over time illustrates the formation and destruction of the film. After 24 h, there is a sudden drop in *R*_f_, which indicates the destruction of the generated film. This drop in the *R*_f_ in the 500Ce-500TSP sample is milder than in other samples and, at all times, the coating resistance of this specimen is higher than the others. Therefore, it can be concluded that a potent compact anti-corrosion film is formed on this sample. On the cathodic sites, the cerium hydroxide/oxide can precipitate due to the Ce^3+^ reaction with OH^−^ ions^38^. Besides, the nitrate and phosphate ions prevented the anodic iron dissolution reaction. The oxygen atoms in the phosphate can share the pair electrons with the empty cerium orbitals and make Ce-TSP complexes^39^. Each cerium can bond with three phosphates and vice versa. The mechanism of the formation of the complex between the cerium and phosphate ions and then the creation of a uniform film without cracking on the sample surface in the presence othese two inhibitors is schematically shown in Fig. 6. So, outcomes are demonstrated that the attendance of the mixture of 500 ppm cerium nitrate with 500 ppm TSP, the increment in the *R*_ct_ and *R*_f_ of the steel was most pronounced among all types of mixtures.

The inhibition efficiency (*η*) was computed via Eq. (2) and, the results are presented in Tables 3 and 4^40^.

2 $$\eta \left( \% \right) = \left( {\frac{{R_{t} - R_{t}^{\prime } }}{{R_{t} }}} \right) \times 100$$

where *R*_t_ is the total resistance of the blank sample and *R*_t_^′^ is the total resistance of inhibited specimen. According to Tables 3 and 4, it is clear that 1000 Ce, as well as 1000 TSP, had the lowest efficiency among all samples. The highest efficiency (around 73.7%) belonged to the 500Ce-500TSP specimen, which is agrees with the polarization test outcomes.

**Figure 7:**
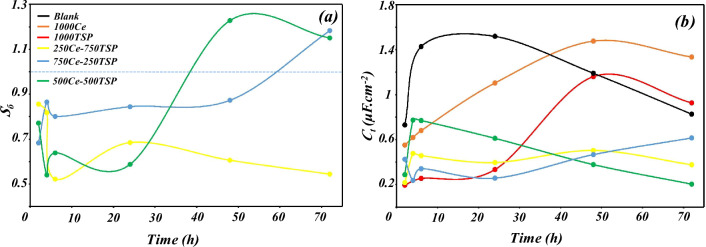
(**a**) Synergism index and (**b**) Inhibition efficiency variations of various samples over immersion time.

Furthermore, the synergism index (*S*_б_) was calculated using Eqs. (3) and (4)^41^ (Fig. 7a):3

4 $$\theta_{{{1} + {2}}} = \left( {\theta_{{1}} + \theta_{{2}} } \right) - \theta_{{1}} \theta_{{2}}$$

where *θ*_1_ and *θ*_2_ are the surface coverage of inhibitors 1 and 2, respectively, and *θ*_1+2_ is the surface coverage in the sample containing 1 and 2 inhibitors. The surface coverage is the percent of the inhibition efficiency (*θ* = *η*/100). The synergism index is depicted in Fig. 7a . The synergism effect occurs when S > 1, and S < 1 is a sign of an antagonism effect^42^. Antagonism happens when the mixing effect of the inhibitors is less than the sum of the sole inhibitors^43^. A high synergism index of the 500Ce–500TSP sample (especially in long immersion times) indicates well synergism effect in this type of mixing and its good corrosion inhibition impact on the steel in the 3.5 wt.% NaCl solution.

In addition, the total thickness of the deposited film (*t*_f_ + *t*_dl_) over time in the various samples was evaluated through the total capacitance, which is drawn in Fig. 7b. The thickness is inversely related to the capacitance. As it turns out, in the 500Ce-500TSP specimen, a thick film has been generated (relative to the film formed in the blank) that becomes higher over time.

### Surface characterization

#### SEM/EDS analyses

**Figure 8:**
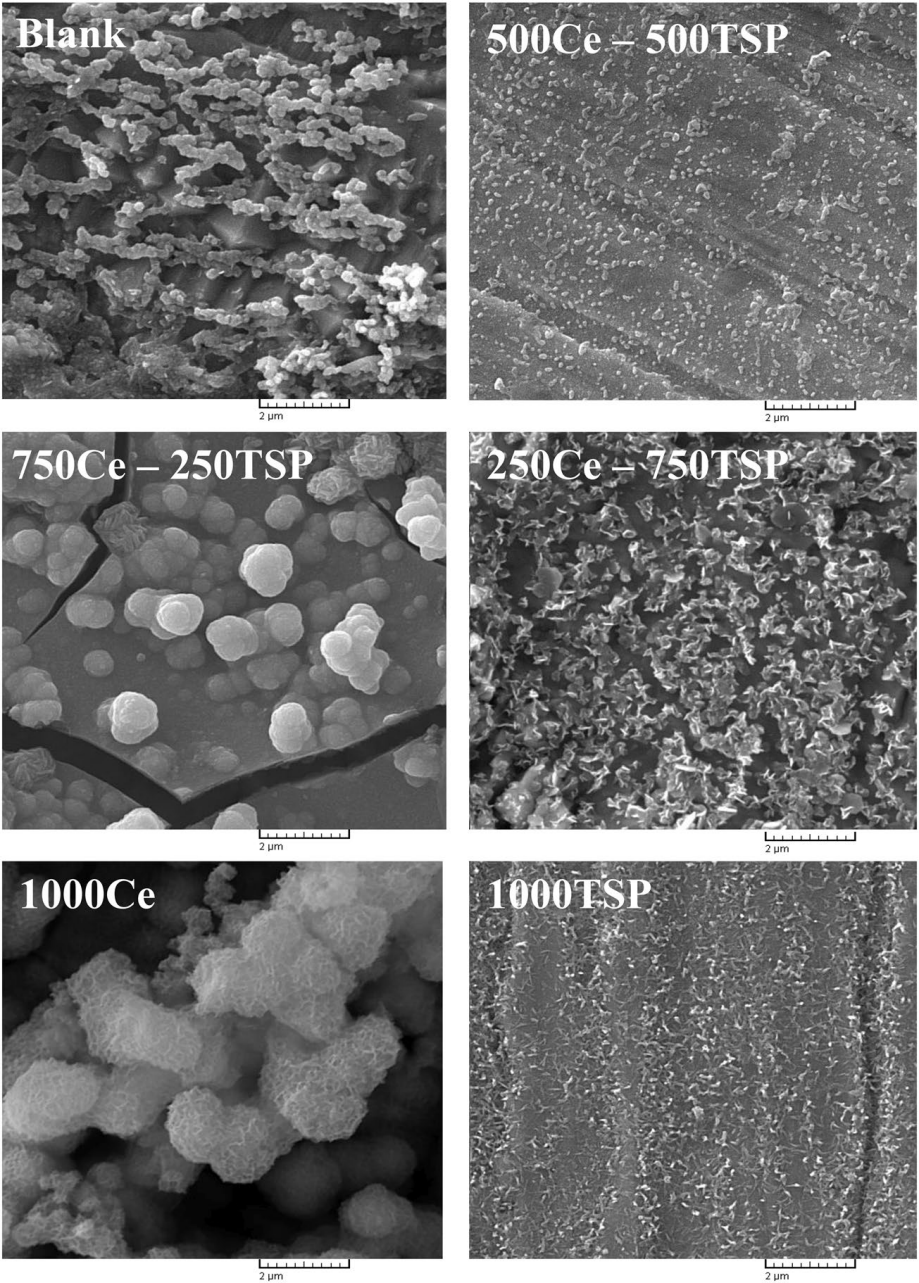
SEM images of the steel samples surface subjected in the 3.5 wt.% NaCl solutions without and with various concentration of Ce/TSP.

The SEM analysis was done to examine the morphology of the immersed steel samples in the 3.5 wt.% NaCl solution with/without inhibition. The results are shown in Fig. 8. In the blank sample, a non-uniform porous layer of corrosion products is formed. In the 1000TSP sample, a thin film owing to the reaction between phosphate anions and iron cations covered the metal surface. In 1000Ce and 750Ce-250TSP samples, a film with deep and large cracks has been created. These cracks are caused by excessive growth of the inhibitor layer and severe film shrinkage during drying. Cracks are easy ways the help the corrosive species reach the steel surface faster and significantly reduce their inhibition efficiency. On the other hand, in 250Ce-750TSP and 500Ce-500TSP samples, a uniform film without cracks can be seen. Cerium cations can form complexes with phosphate ions and, or form Ce-oxide/hydroxide on the metal surface through a reaction with OH^−^. In the equal ratio of the inhibitors, a film is generated with better properties than the 250 ppm cerium nitrate + 750 ppm TSP ratio. The formation of the complex is probably the leading cause of producing a protective film with the highest compaction.

**Figure 9:**
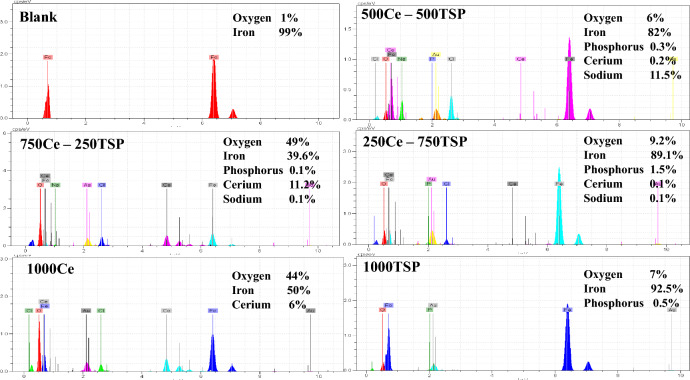
EDS spectrum of the steel samples surface subjected in the 3.5 wt.% NaCl solutions without and with various concentration of Ce/TSP.

In order to analyze the surface film composition, EDS was accomplished. The relevant results are catered in Fig. 9. The high quantity of O and Fe in the uninhibited specimen was potent evidence for the corrosion reaction product growth on the surface. The generation of a layer of cerium in the 1000Ce sample is responsible for the low content of Fe in this specimen. The 1000TSP sample has the highest percentage of Fe among the protected samples, revealing the low surface coverage. In the samples immersed in a mixture of inhibitors, both of the inhibitors partake in the film-forming process, as proved by the detection of a high value of cerium and phosphorus elements on the surface.

#### Contact angle test results

**Figure 10:**
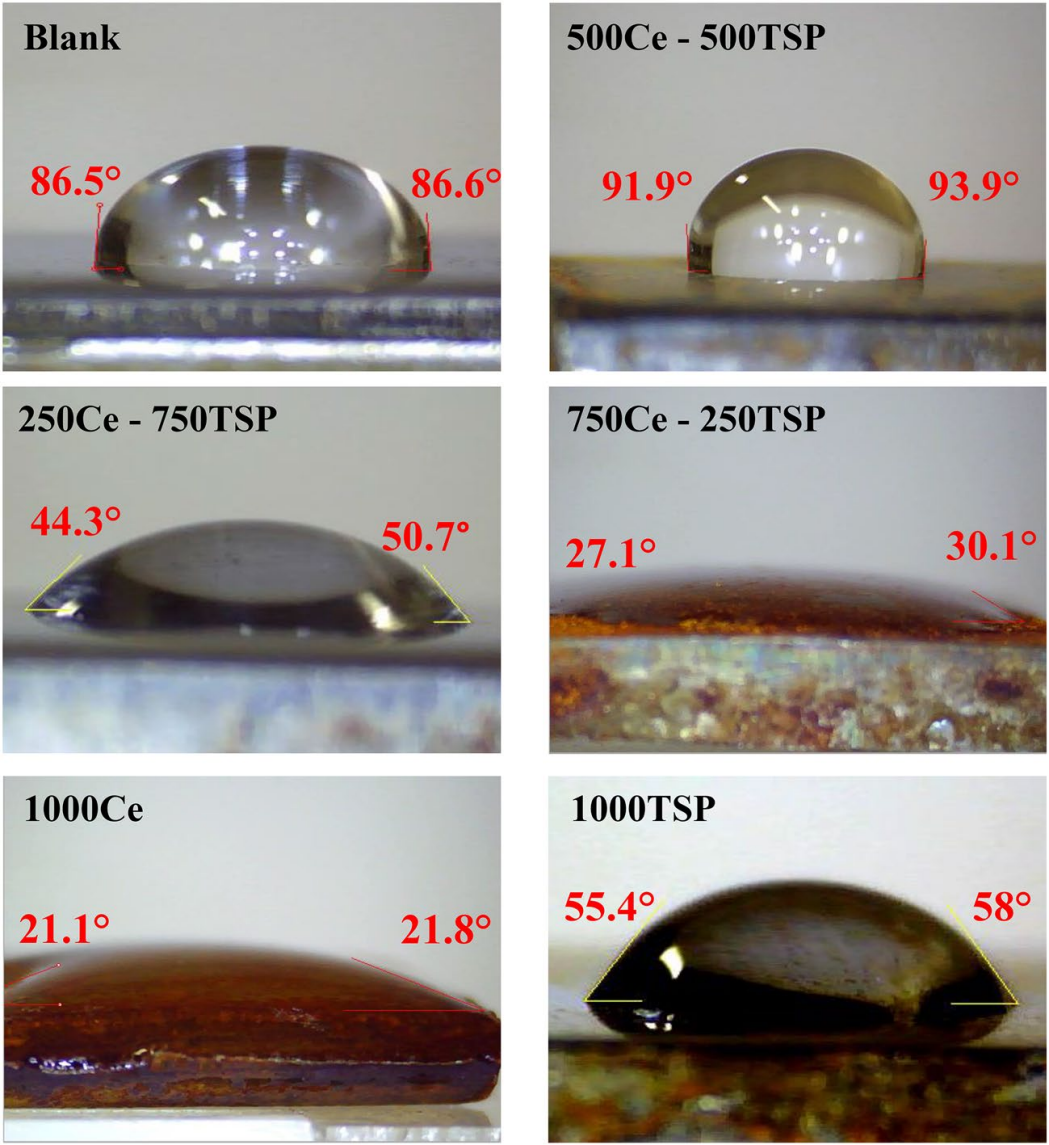
The contact angel of DW on the surface of various samples immersed in the 3.5 wt.% NaCl solutions without and with various concentration of Ce/TSP for 48 h.

In order to check the γ (surface free energy) of the samples covered with the inhibitory film, the CA test was utilized^44^. The contact angle test of the samples was performed after 48 h of immersion in relevant solutions using DW. The appearance of the samples, along with the contact angles created by the water droplet with the substrate, is shown in Fig. 10. The *θ*, *W*_A_, and *γ*_sv_ (calculated via Young’s equation & Neumann’s formula^45^) values of various samples are reported in Table 5. The blank sample surface is hydrophilic because of the vast amount of corrosion products that covered the surface, as observed in the SEM micrographs. The hydrophilic nature of the samples containing cerium cation was related to forming a layer of the hydrophilic compounds i.e., cerium hydroxide, on the steel surface. Among all samples subjected to the mixture of inhibitors, the highest contact angle and lowest surface energy were recorded for the 500Ce-500TSP specimen. The surface hydrophobicity of this sample can be one of the reasons for its good corrosion inhibition due to its low wettability properties.

**Table 5:** Contact angle, work of adhesion and surface free energy values of various samples.

Sample	*θ* (°)	*W*_A_ (mJ/cm^2^)	*γ*_sv_ (mJ/cm^2^)
Blank	86.6	77	31
1000Ce	21.5	140	68
1000TSP	56.7	112	49
750Ce-250TSP	28.6	136	65
250Ce-750TSP	47.5	121	55
500Ce-500TSP	92.9	69	27

*θ* contact angel, *W*_A_ work of adhesion, *γ*_SV_ surface free energy.

#### XRD analysis

**Figure 11:**
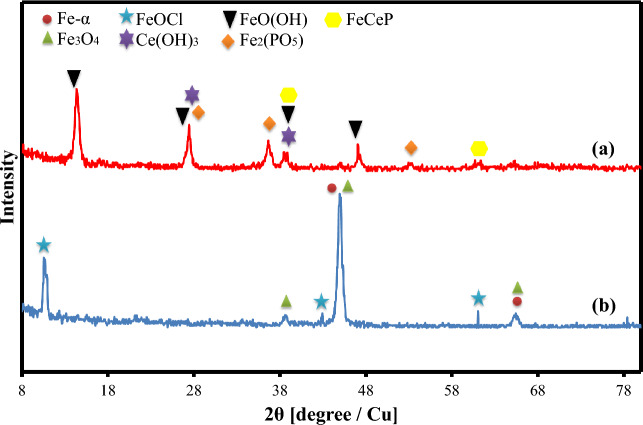
XRD spectrum of the (**a**) Blank and (**b**) 500Ce-500TSP samples.

In order to investigate the inhibitory film and its phases, the XRD test has been employed. The results of this test are shown in Fig. 11. In the blank sample; some phases have been identified, such as iron (as the substrate's main element) at 2θ = 45°, Fe_3_O_4_ (due to the corrosion of the substrate in the saline environment) at 2θ = 39°, and FeOCl at 2θ = 11°. In the XRD spectrum of the 500Ce-500TSP specimen, some phases are distinguished. Cerium-hydroxide and iron-phosphate as main phases prove the reaction of inhibitors and complex formation which was also confirmed through previous tests. Furthermore, some sub-phases have been identified in the inhibitory film with Fe, Ce, P, O, and H elements that admit the inhibitors' reaction with the substrate. In addition, the absence of the Fe_3_O_4_ phase (corrosion products) and Fe in the 500Ce-500TSP spectrum can be related to the presence of the thick and dense inhibitory film. The SEM/EDS analysis results also vouched for this claim.

#### FT-IR spectroscopy

**Figure 12:**
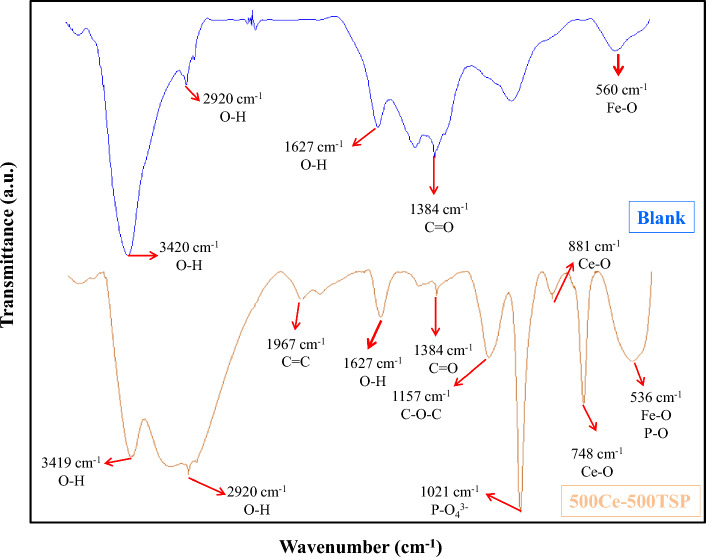
FT-IR test results of the Blank and 500Ce-500TSP samples.

FT-IR test was conducted to determine the composition of the inhibitory film. The result of this test is given in Fig. 12. In the blank sample; there are some peaks in 560, 2920, and 3420 cm^−1^ that they attributed to Fe–O and O–H stretching vibration, respectively. In the 500Ce-500TSP sample, many peaks were observed, peaks which appeared in (748 and 881), (536 and 1021) cm^−1^ wavenumbers respectively related to Ce–O and P–O bonds. These bonds can prove the presence of the cerium-hydroxide and cerium-phosphate on the inhibited steel substrate (Fig. 6). Also, the 536 cm^−1^ peak may be attributed to the Fe–O bonds related to Fe–OH and Fe-phosphate phases in the inhibitory film (Fig. 6). Some peaks in 1627, 2920, and 3420 cm^−1^ can appertain to O–H bonds. In addition, other peaks i.e., 1967, 1384, and 1157 cm^−1^ represent C=C, C=O, and C–O–C bonds, respectively, which can be attributed to impurities. These results, confirm the presence of Ce and P elements in the composition of the inhibitory film, which is the result of the reaction of two inhibitors and the complex formation. It’s worth mentioning that the presence of Ce and P elements in the protection layer was confirmed by the EDS test, and the attendance of phases consisting of Ce and P elements in the protection layer was also proved in the XRD test^46–48^.

## Conclusion

In this study, the application of cerium(III) nitrate and phosphate anions toward inhibiting the mild steel corrosion in saline media and their synergistic inhibition impact was explored. The best combination (with the highest inhibition efficiency) between tri-sodium phosphate (TSP) and Ce(III) ions was determined as 500 ppm Ce(III) nitrate beside 500 ppm TSP (500Ce-500TSP) through electrochemical tests. Corrosion assessment was performed using EIS and the potentiodynamic polarization technique. In addition, the SEM, EDS, CA (contact angle), XRD, and FT-IR tests were done to inspect the deposited film morphology and composition.

1. The electrochemical test confirmed that the 500Ce-500TSP sample produced a compact film on the steel sheet that caused better corrosion resistance of this specimen than the others. The *R*_ct_ of this sample was more than 7 kΩ cm^2^ (fourfold more than the blank sample). The inhibition efficiency was calculated by EIS outcomes for all samples and, it revealed that the 500Ce-500TSP sample had been destroyed with 79.7% efficiency (more than other specimens).
2. In SEM/EDS test results of the 500Ce-500TSP sample, a uniform film without cracks was observed owing to the formation of organic/inorganic complexes. In EDS analysis of the 500Ce-500TSP sample, the high value of cerium and phosphorus elements on the surface was obtained, confirming the complex formation of inhibitors on the surface.
3. Among all samples subjected to the NaCl mixture of inhibitors containing media, the highest contact angle (92.9°) and lowest surface energy (27 mJ cm^−2^) were recorded for the 500Ce-500TSP specimen. The surface hydrophobicity of this sample can be related to the presence of cerium hydroxide, which causes good corrosion inhibition and low wettability properties.
4. XRD and FT-IR analysis investigations revealed the composition of the inhibitor film. As a result of these tests, the presence of two inhibitors in the form of the main phases i.e., cerium hydroxide and iron phosphate, are proved. These tests can confirm the organic–inorganic complexes' formation on the inhibited steel surface.

## Data Availability

All data generated or analysed during this study are included in this published article.
